# Creating an Enabling Political Environment for Health and Social Care Integration

**DOI:** 10.5334/ijic.2531

**Published:** 2016-10-28

**Authors:** Anne Hendry

**Affiliations:** Joint Improvement Team, Scottish Government Health and Social Care Directorates, GB

## Introduction

The Scottish Parliament recently passed legislation on integrating health and social care in Scotland. In this perspective paper, the clinical lead who supported the development and implementation of national policy on older people, long term conditions and integrated care in Scotland describes how political, policy and professional leaders have together created the right conditions to enable this ambitious change. The author reflects on the respective contributions from innovation, improvement, co-production, financial incentives, and through a clear focus on outcomes for people – whether patients, clients, carers, staff or citizens. The paper discusses how Scotland adapted Kotter’s eight steps for managing change and explores the transferable learning for other regions embarking on system transformation for integrated care.

## What did we do and why?

Like most developed countries, the population of Scotland is increasing and ageing – projected to rise by seven per cent to 5.7 million between 2014 and 2039 with an 85% increase in the population over 75 years [1]. Already 34% of households include at least one adult or child with a long-standing illness, health problem or disability and this figure rises to 45% in lower income households [2]. Analysis of data on the demographic change and the anticipated demand led to widespread recognition that the current system is neither desirable nor sustainable. I believe that analysis created our burning platform.

Scotland has fourteen NHS Health Boards responsible for planning and delivering hospital, primary care and community services for their local populations. The NHS Boards work closely with 32 local authorities that directly provide or commission social care and housing services from the independent and Third sectors. Integrated care features in most healthcare policy documents [3,4] yet Audit Scotland, the independent public spending watchdog, reported few good local examples [5] and it is widely acknowledged that progress on integrated care has been too slow. Service integration was also a priority in the Christie Commission Report on the Future Delivery of Public Services [6]. I believe the cross government support for public service reform influenced the decision by all major political parties to include a commitment to integrate health and social care in their Manifestos for the 2011 Scottish elections [7]. With the Scottish National Party winning a second term, this time with a majority in the Scottish Parliament, the Scottish Government embarked on legislation to integrate health and social care.

The Public Bodies (Joint Working) (Scotland) Act (2014) requires the local integration of adult health and social care services with the option to include children’s health and social care services, criminal justice social work and housing support services in local integrated arrangements [8]. The Act requires NHS Boards and Local Authorities to establish one of two models by April 2016: delegation of functions and resources between Health Boards and Local Authorities (Lead Agency), or delegation of functions and resources to a Body Corporate (Integrated Joint Board). Each Integration Authority will oversee an integrated budget for agreed functions. The sum of these integrated budgets will be more than £8 billion (more than 60%) of health and social care resources, including all adult social care, adult primary and community health care, and those aspects of adult hospital care that are most amenable to redesign through enhanced primary and community care.

A strategic plan and integrated budget, developed with involvement of providers, non-statutory partners, patients, carers and service-user representatives, will commission the required range of integrated services and community support to improve local population health. Progress will be measured against nine health and wellbeing outcomes supported by a suite of 23 indicators that track care experience and data on activity and resources.

## Creating the Conditions to get there

Legislation is not a quick fix, even when the conditions are favourable as they were in Scotland. This was more a marathon than a sprint. The carefully timed and considered journey took almost three years from announcement of intent to enacting the legislation. Officials reviewed the evidence on successful integrated systems to identify key concepts to include in the legislation: plan for the needs of local populations, pool resources (money and people), involve care professionals in service planning, investment and delivery, and ensure strong local leadership and accountability. Extensive engagement with health and social care leaders, professional organisations, staff and local communities took many months but I believe this was an investment that was critical for success. The approach involved much listening and many conversations across the country. The dialogue was consistently framed around a vision to improve outcomes: people should be supported to live well at home or in the community for as much time as they can and should have a positive experience of health and social care when they need it [9]. When Audit Scotland reported widespread support for the principles of integration [10], I viewed that as a testament to effective engagement that involved people from all sectors and used both data and personal narratives to secure commitment at all levels.

The path to legislation had strong leadership from a Ministerial Strategic Group for Health and Community Care, chaired by the Cabinet Secretary for Health and Wellbeing. This high level group of local political and NHS leaders was supported by senior policy officers, professional leaders and technical experts on issues such as governance, finance and outcomes. This Ministerial group predated the focus on integration and had established mutual trust and effective cross sector relationships through their previous work to oversee the Reshaping Care for Older People (RCOP) programme [11]. This programme required formal, albeit voluntary, collaboration between local health, social care, housing, voluntary and independent sector partners. I believe this was a critical step that moved health and social care integration from the abstract to the local living lab. I had the privilege of drafting an overview report on the valuable action learning that nurtured collaborative behaviours and built organisational readiness for integration [12]. The clear line of sight between policy, delivery and practical support for improvement was another important enabler. The Joint Improvement Team, with cross sector expertise in adaptive improvement, facilitated an improvement network to support local teams to spread good practice, tackle variation and track progress on a core set of improvement measures.

I consider operating in financial austerity as both a challenge and an opportunity. A £300 million Change Fund (around 1% of the older people health and social care budget) was introduced over four years from April 2011. Funding was ring fenced to test and spread interventions to enhance the wellbeing and independence of older people and their carers, prevent, reduce or delay dependency, improve experience and personal outcomes, and increase the resilience of the system. Early wins are critical to secure buy in and continued investment in new ways of working. The *Reshaping Care and Change Fund Building on Progress Report* (12) contains over 100 case study examples and evidences the shift in national indicators – e.g. a 10% reduction in rate of emergency admissions of over 75s; older people spent around 2.5 million more days at home in 2014/15 than would have been expected.

Learning from what worked for RCOP is now being applied to all adult care groups through an Integrated Care Fund of £300 million over three years from April 2015. Guidance on the use of the Integrated Care Fund [13] encourages investment in preventative supports and acknowledges the crucial role of the voluntary sector in supporting the assets of individuals and communities so that people can have greater control over their own lives and develop capacity and confidence in self-management. Additional investment in social care, telehealth and telecare, primary care, and mental health services will also help to build capacity in community services [14].

## Lessons for other systems

The conditions in Scotland are set for a unique and large scale transformation in health and social care. It has been a complex and challenging journey but the April 2016 milestone for integrated service planning and budgets is simply the end of the beginning – and the start of an exciting phase of local transformation. I believe our success factors are having a consistent policy direction with strong cross government leadership; a narrative on improving outcomes for people through valuing the assets of local communities and embracing genuine coproduction; and building organisational readiness, commitment and evidence of what works through the RCOP programme and Change Fund.

There is no easily transferable blueprint for integrated health and social care. Understanding complexity, context and culture are critical for creating the right conditions. We did not adhere to a detailed route map but I believe the change approach we adopted largely reflects John Kotter’s eight steps [15]. This paper reflects my perspective on our progress in the first seven steps. Now we face the most difficult step – to stick with it for the long haul!

Looking forward, I consider our greatest challenge will be in transforming our workforce. The workforce of the future is here now – in our schools, colleges, universities and health and care organisations. We need to empower people to work in new ways that are person centred, integrated, engage the local community and enable participation by the people who use support and care in the community in which they live.

I believe that we must – and we will – stick with this change. Because the alternative is no longer sustainable – and it’s not the future that I want.

## Author Information

Anne Hendry is a consultant geriatrician, Honorary Professor at the University of the West of Scotland, and worked with the Scottish Government and Joint Improvement Team as clinical lead for Integrated Care. She continues to support professionals to improve outcomes for older people and people with multiple conditions in Scotland through person-centred care and support that fully integrates health, social care, housing, community and voluntary sectors, and is co-designed and delivered with people in local communities. Anne recently became a Senior Associate with the International Foundation for Integrated Care.
